# Design and implementation of a highly accurate spatiotemporal monitoring and early warning platform for air pollutants based on IPv6

**DOI:** 10.1038/s41598-022-08416-5

**Published:** 2022-03-17

**Authors:** Rongjin Yang, Xuejie Hao, Long Zhao, Lizeyan Yin, Lu Liu, Xiuhong Li, Qiang Liu

**Affiliations:** 1 State Key Laboratory of Environmental Criteria and Risk Assessment, Chinese Research Academy of Environmental Sciences, No. 8, Da Yang Fang, An Wai, Chao Yang District, Beijing, 100012 China; 2 State Key Laboratory of Remote Sensing Science, College of Global Change and Earth System Science, Beijing Normal University, No.19, Xinjiekou Wai Street, Haidian District, Beijing, 100875 China; 3 Higher Institute of Computer Modeling and Their Applications, Clermont Auvergne University, Clermont Auvergne, France

**Keywords:** Climate sciences, Environmental sciences

## Abstract

The development of industry has brought about the pollution of the atmospheric environment. Pollution is harmful to people's health. Realizing the real-time monitoring of atmospheric environmental quality parameters can improve the above-mentioned effects. China's existing environmental monitoring systems focus on the accuracy of the system hardware itself for assessment, lack of data analysis and forecasting and early warning, and cannot provide managers and ordinary people with decision-making and activity guidance. This paper develops an IPV6-based high-spatial–temporal precision air pollutant monitoring and early warning platform. The feasibility of the system is verified through networking tests, operation tests, and early warning tests. Through actual data analysis and comparison, it is concluded that the monitoring system has field feasibility, and the atmospheric environment monitoring for the target observation area has achieved the desired observation function. This system integrates GIS technology and B/S architecture to analyze changes in the regional environment to provide support for regional environmental air quality management. The forecast and early warning module constructed by combining the weight method of the influence of different input factors on the environmental quality index and minute-level observations can provide technical support for the government to improve the level of supervision.

## Introduction

In recent years, environmental pollution has become more and more serious. Realizing real-time monitoring of atmospheric environmental quality parameters and making predictable decisions are important issues that need to be resolved. At present, domestic atmospheric environment monitoring is mainly achieved by using traditional methods of state-controlled sites and provincial-controlled sites, which requires a lot of manpower and material resources, high cost and low efficiency, especially for China, which is a vast region, where sparse sites cannot achieve precise monitoring. How to achieve regional atmospheric environmental monitoring with high temporal and spatial accuracy, realizing real-time physical examination of regional atmospheric environmental quality monitoring, and improving the level of regional air quality management and decision-making is a key research topic.

Research and development of environmental monitoring systems across the world mostly focus on hardware components, while the software platform is only considered an auxiliary component with a single function that only focuses on the production of simple data visualization. There is a lack of applied research on advanced data analyses, predictions, and early warnings, and the existing research mainly focuses on aquaculture, deformation monitoring, and water quality monitoring.

In 2020, Chien Lee developed an Internet of Things monitoring system for metabolism and aquaculture fish activities based on cloud computing. The system collected minute-level data through wireless sensors, established communication connections between wireless sensors and central servers, uploaded data in real-time, and conducted regression analyses on data in real-time with a cloud server program, thus forecasting and providing warnings of the health status of the fish being studied^1^. Katsuda et al. developed a deformation monitoring system based on a wireless sensor network in 2019. The wireless sensor of the system adopted a modular design and integrated a high-precision ranging module to monitor wall deformations. The sensor provided visual online previews of data on the Web through computer graphics technology and Web networking technology^2^. In 2017, Ziran Zhang used water balance sensors to measure changes in snow, soil humidity, air, temperature, and humidity of a river basin in the United States and developed a complete front-end system to facilitate data storage and retrieval. The system established communications with a central server through the wireless network module of the sensor, and the central server stored the data received in real-time on a local server. The background managers could log in to the background database to view the historical data and conduct analyses. The whole system included the design of the wireless sensor, the establishment of network communications, and the design and implementation of the background database system but lacked complex background data visualization and analysis functions^3^. In 2019, Demetillo, Alexander T, and others presented a large-scale water quality monitoring system based on wireless sensor network technology, which transmitted collected data to a global mobile communications module and forwarded it to a preselected mobile phone number in the form of a short message^4^. Additionally, in 2019, Salman, N, and others presented a real-time indoor air quality monitoring system based on a wireless sensor network. The system was connected to the central base station through a ZigBee module so that users could monitor the data from the wireless sensors online and in real-time and extend the point data to the area displayed through kriging interpolation^5^. The comparison of advanced technologies is shown in Table 1.

**Table 1:** The comparison of advanced technologies.

Representative author	Contribute	Result	Application field
Chien Lee	ChienLee has developed a cloud-based aquaculture fish metabolism and activity IoT monitoring system	The system predicts and warns the health of fish	Aquaculture
Katsuda Y	Katsuda Y has developed a deformation monitoring system based on a wireless sensor network	The system monitors the deformation of the wall and provides a visualized online preview of the data on the website	Architecture
Ziran Zhang	Ziran Zhang uses a water balance sensor to measure changes in snow, soil moisture, air, temperature, and humidity in a river basin in the United States and has developed a complete front-end and back-end system	The system stores the data received in real-time to the local server, and managers can view historical data and make corresponding analyses	Waters
Demetillo	Demetillo proposed a large-scale water quality monitoring system based on wireless sensor network technology	The system forwards the collected data to the pre-selected mobile phone number in the form of the short message	Waters
Salman, N	Salman, N proposed a real-time monitoring system of indoor air quality based on a wireless sensor network	Through this system, users can understand the monitoring data online in real-time	Air

Currently, to forge a connection between individual things and the Internet, it is necessary to assign an IP address to each sensor^6^, which, for environmental monitoring systems, requires a large number of IP addresses. However, the total space available for IPv4 addresses across the world is only 256B. As of November 25, 2019, the European Network Coordination Center announced that all IPv4 addresses have been allocated. If IPv4 technology continues to be used, the deployment of sensor networks will face huge problems due to the shortage of IPv4 addresses^7^. Therefore, an alternative method is to embed the IPv6 protocol into sensors by using transitional technology. IPv6 has a larger address space of 232 bits, which enables up to 2^128^ addresses, meeting the requirements of a large-scale and high-density sensor layout.

According to the 45th Statistical Report on Internet Development in China, the number of IPv6 addresses in China is 50,877/32, which ranks China first in the world. However, IPv6 still has some problems, such as lack of development and insufficient popularization. At present, the IPv4 network is still dominant in China, and it is almost impossible to upgrade to an IPv6 network in a short time because of the large manpower and material resource requirements. Therefore, upgrading from IPv4 to IPv6 primarily uses transitional technologies and gradually introduces IPv6 technology^8^. The RFC 1933-Transition Mechanism for IPv6 Hosts and Routers, published in 1996, is the first standardized IPv6 transition technology. This technology operates on the principle that an IPv6 packet is a specific payload of an IPv4 packet. This method can only be used in P-to-P scenarios, and the tunnel exit must be manually configured, which is cumbersome, causing it to not be widely adopted. RFC 2473-Generic Packet Tunneling in the IPv6 Specification is the second standardized IPv6 transition technology^9^, and its implementation mechanism is the same as that for RFC1933. In 1999, the third standardized IPv6 transition technology, RFC2529, was presented and is referred to as 6over4 technology for short. Subsequently, from 2006 to 2007, IPv6 transition technologies such as RFC4798 and RFC4659 were introduced one after another; they could automatically configure tunnel exits, and these technologies were sought after by global network operators, such as AT&T in the United States, FT in France and TI in Italy^10^.

Currently, China's environmental pollution is very serious, and its poor air quality is especially worrying^11^. All regions in China have different degrees of air pollution. Monitoring of the atmospheric environment is mainly done by monitoring the concentration index of atmospheric pollutants. Traditional atmospheric conditions are measured in real-time using monitoring stations, but they have significant limitations. First, the cost of building monitoring stations is relatively high, and there are very few monitoring station installations; therefore, it is difficult to generate accurate monitoring results over a wide region^12^. Wireless sensor networks are more convenient, and they are relatively low in cost and easy to develop and deploy. This paper introduces an air pollutant monitoring and early warning platform with high space–time accuracy based on IPv6 technology. Our platform solves the problem of IPv4 address shortages by using IPv6 technology^13^. The proposed platform has rich data visualization functions for analyzing air pollutant conditions and makes use of deep learning algorithms to predict and warn of air pollutant concentrations based on historical data. The platform solves the limitations of the existing technology as shown in Table 2.

**Table 2:** The platform solves the limitations of the existing technology.

Limitations of traditional monitoring systems	Does this platform solve this limitation
High cost	Yes
The number of monitoring sites is large	Yes
Complex development and layout	Yes
Unable to achieve regionalized monitoring	Yes

The novelty of this research is to use high-temporal precision data to monitor the regional atmospheric environment, combined with the current advanced computer technology, to realize the online operation of regional atmospheric environment monitoring, and to realize the real-time data of regional environmental monitoring with the high-precision collected data. Preview and analyze, integrate GIS technology and B/S architecture to analyze changes in the regional environment, and provide support for regional environmental air quality management. The relationship between the atmospheric environmental quality and the observed atmospheric factors was analyzed and compared with the collected high-temporal-spatial-accuracy data, and the influence weights of different input factors on the environmental quality index were analyzed and compared. Utilizing the high time accuracy of the wireless sensor network collection feature, the minute-level change observation of the atmospheric environmental quality of the target observation area is realized. Using this feature, timely analysis and alarms can be made on the changes in the atmospheric environment quality of the target observation area. The realization principle is realized by configuring early warning rules. It is judged whether the collected data meets the early warning rules through timeliness. If the early warning rules are triggered, the system Quickly sends the information to the involved units and relevant responsible departments to provide technical support for the government to improve the level of supervision. Through multi-dimensional data analysis, scientific evaluation of the key factors affecting the air quality in the control area is carried out.

This paper develops an IPV6-based high-temporal-accuracy air pollutant monitoring and early warning platform. The system is realized by a combination of software and hardware. The hardware platform adopts a modular design scheme with embedded IPV6 protocol to solve the problem of IP address shortage. The software platform is responsible for data reception, combined with GIS technology to realize the visualization of regional atmospheric environmental data and provide data early warning, data trend analysis, and other functions.

## Overall design of the system platform

The system consists of two parts: software and hardware platforms^14^. The software platform is a functional module based on a B/S architecture that conducts data acquisition, data analysis and processing, data visualization, data trend analysis, air pollutant interpolation analysis, and data early warning. The hardware platform is a wireless sensor network, which includes a motherboard and data acquisition board.

The software operating environment is a Web portal server with 4 CPUs, 8 GBs of memory, 160 GBs of disk, and the 2012 Windows Server operating system. The software environment has a running environment that uses JAVA 8, Apache Tomcat 9.0.8, MySQL, and other software preinstalled on the server.

### Hardware platform design

Since the platform is a sensory platform, the hardware system adopts a modular design, which was mainly applied to the design and development of the motherboard and data acquisition board. With an ARM processor as the core, the IPv6 protocol is embedded in each sensor node to operationalize the data acquisition and upload functions.

#### Motherboard

The motherboard is the core component of a wireless sensor, and it is composed of a charging management unit, power conversion unit, clock management unit, network interface, J-Link component, data storage unit, network interface, and signal transceiver. With the STM32F107 chip as the core, the IPv6 protocol is embedded to control the acquisition and storage of data in field environments and is responsible for the communication between wireless sensors and servers.

#### Data acquisition board

**Figure 1:**
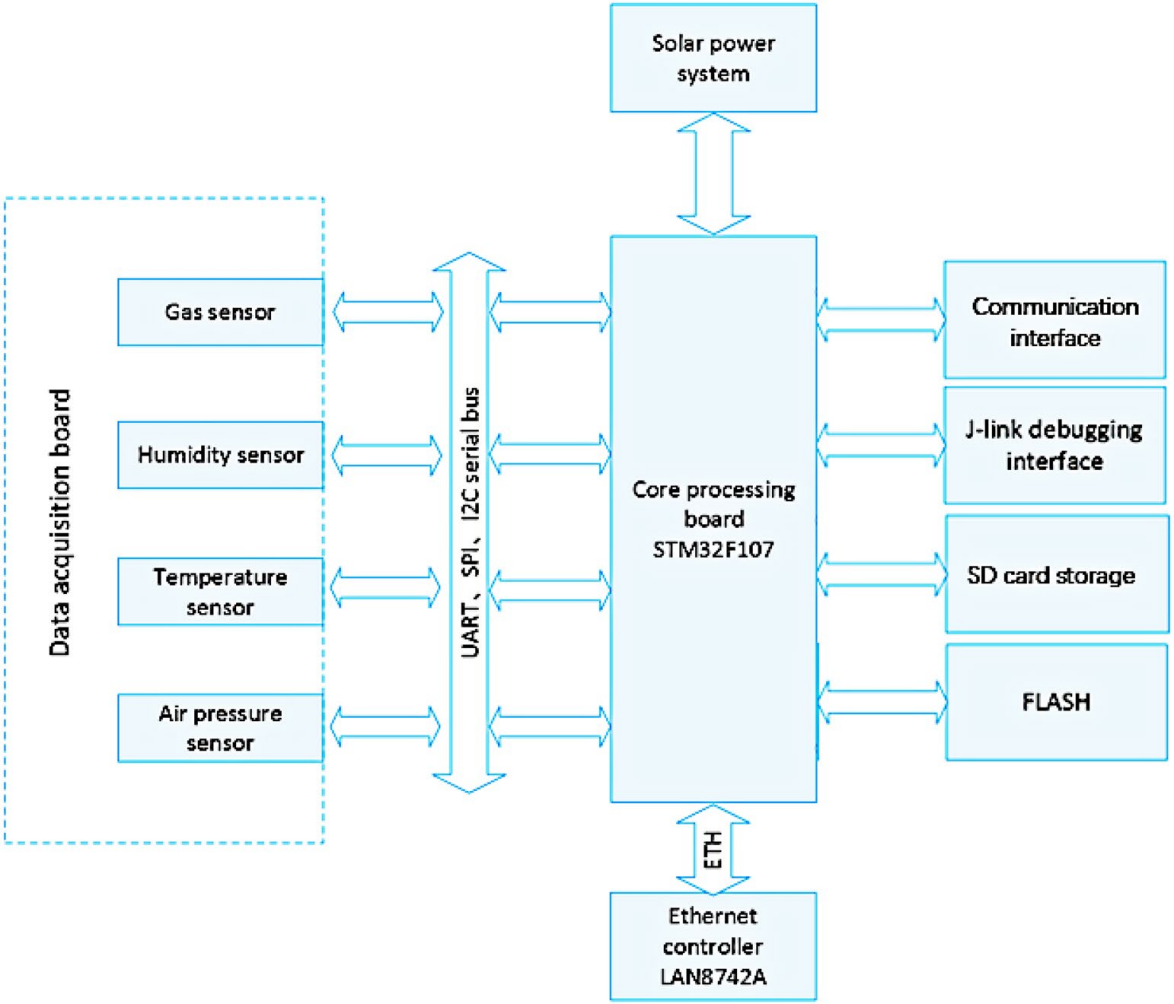
The entire hardware framework.

Air quality is monitored by a series of air pollutant concentration indexes, so equipment must be used to connect different sensors and equipment^15^. For different sensor types, the acquisition board requires various connection modes to interact with the motherboard. Therefore, the data acquisition board is separated from the motherboard to improve the universality and scalability of the whole system. The separately designed digital acquisition board connects to each type of sensor. For example, a digital sensor is the communications interface between the sensor and the processor provided by the data acquisition board; an analog sensor is a functional component provided by the data acquisition board to connect the final amplified or converted data with the processor when the sensor signal needs to be amplified and converted, and the output pulse sensor is used to shape the pulse signal output by the sensor. The I/O ports on the motherboard include 12 A/D converters and 12 D/A converters, as well as synchronous and asynchronous transceivers, a controller area network, timers, counters, and internal integrated circuits^16^. The entire hardware framework is shown in the following Fig. 1.

On the platform, the data acquisition board is shown in Fig. 1. Through this board, the sensor can communicate with the mainboard and provide data interaction. For the above-mentioned different types of sensors, the interaction between the data acquisition board and the mainboard is different. For example, for digital sensors, the data acquisition board only provides an interface for these sensors to communicate with the motherboard. For analog sensors, the data acquisition board amplifies and converts the electrical signals output by the sensors, and then passes them to the mainboard for processing through a dedicated interface.

### Software platform design

The overall framework of the monitoring and early warning platform for air pollutants with high space–time accuracy based on IPv6 is shown in Fig. 1. The platform is divided into the data acquisition module, the data analysis, and processing module, and the data visualization and early warning module.

The data acquisition module monitors the status of atmospheric pollutants through wireless sensors, builds a system database, and sends the data on the atmospheric pollutant concentration status to the background server in real-time. The data analysis and processing module are responsible for receiving the message data sent by the wireless sensor and unpacking and analyzing the data according to the specified analysis rules. The data visualization and early warning module is responsible for the front-end display of the data, determines abnormal monitoring statuses, flags unexpected monitoring data from the monitoring points based on the received data, and feeds the information back to the management personnel in a timely fashion.

#### Data acquisition module

The design and implementation of the data acquisition module require a 4G communication network to implement data uploading, and a wireless sensor network collector terminal implements data forwarding through an embedded system. At present, the socket port monitoring data on the server are implemented using the TCP protocol^17^. After the terminal data collector collects data, it requests that the server interface establish a connection with the server to facilitate communication. After the server receives the data transmitted by the collector, it unpacks and analyzes the collected data according to the format of the protocol message. For a specific acquisition factor, it is necessary to analyze the data according to their corresponding analysis parameters. After the analysis is completed, the data are stored in the database to wait for the data call. The specific operation flow is as follows:

1. First, the data are accepted and stored in the local buffer through the socket interface, and the completely agreed message data are accepted
2. Then, the sensor mark position is analyzed corresponding to the data, and the data are converted behind the data position, according to whether the corresponding sensor has data (0 means no data, 1 means data)
3. According to the agreed resolution factor of each acquisition parameter, different parameters are multiplied by different resolution factors, and then, the data-bind with the equipment after the corresponding equipment ID is resolved.
4. To ensure the readiness of the data, the calibration equipment is installed at the near-ground position, and the accuracy of the data collected by the sensors is updated by comparing the collected and analyzed data.

#### Data analysis and processing module

The data analysis and processing module analyze the collected data, and the current TCP connection node is message data obtained through the socket interface. For different acquisition factors, data are arranged according to whether the corresponding data collector has data, and then, the data format is filled after the flag bit. The data bits have already agreed to the arrangement rules in the data analysis part, and each bit represents a different data sensor. To save the stored message length, the collected data are filled after the flag bit; otherwise, they are not filled.

As shown in above Table 3, the collected data, the data flag bits, and the corresponding data bits are analyzed to obtain the corresponding real data.

**Table 3:** The specific meaning of each position of the received data.

Markr bit	Markr bit	…	Markr bit	Data bit	Data bit	…	Data bit	Equipment number	Time and location
0	1	…	1	xxx	xxx	…	xxx		xxxxxxx

To facilitate the transmission of data and to avoid the complexity of data formats caused by decimals, the scaling parameters of each parameter are agreed upon through the parsing program during data acquisition to ensure that the data transmission results are integers. After unpacking and parsing the basic data, the data are post-processed according to the agreed-upon parameters. The corresponding acquisition equipment binds to the data according to the transmitted equipment number.

#### Data visualization

After collecting the high spatiotemporal precision data collected by the wireless sensor network in the server, our main task is to visualize the data to show changes in an atmospheric environment in a useful way for decision-makers.

**Figure 2:**
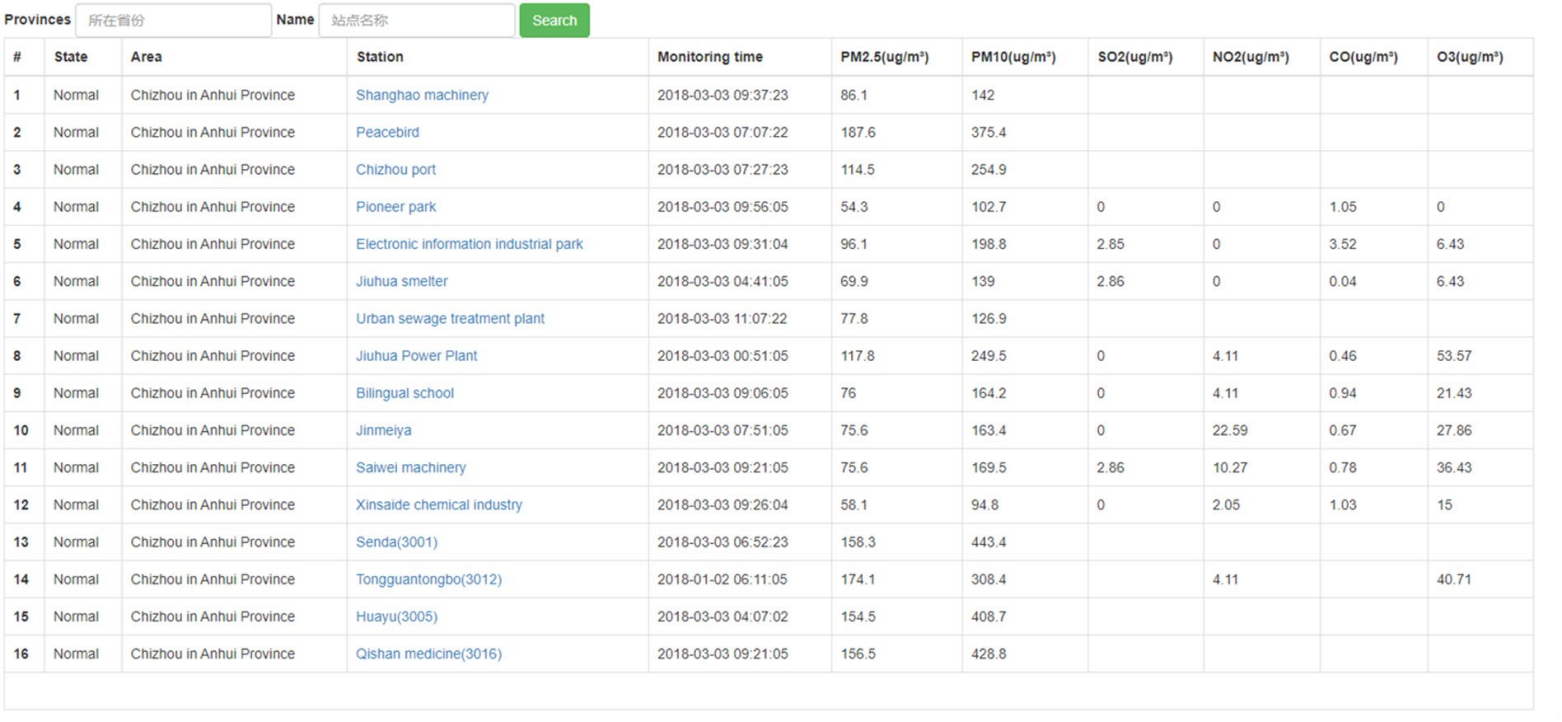
The statistical interface of the latest monitoring data of each site of the monitoring and early warning platform.

The overall architecture of data visualization is shown in the figure below, which is mainly composed of a front-end call, background data processing, and a data storage module. For the background data call process, we use the spring MVC framework in Java, which ensures the implementation of the front-end real-time data display function^18^. Figure 2 shows the statistical interface of the latest data for each site:

##### Data trend analysis and change

**Figure 3:**
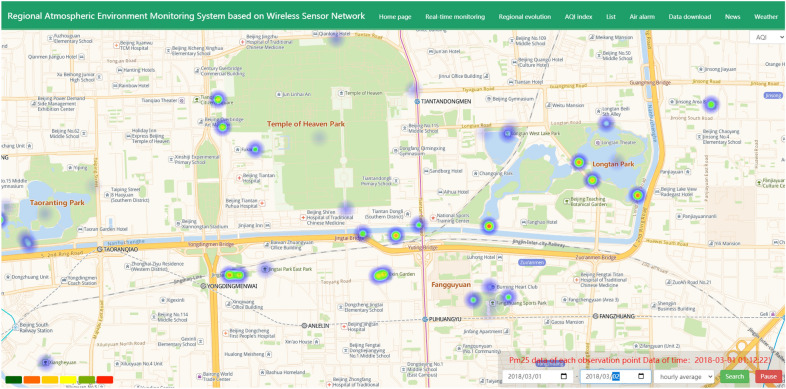
Because of the AQI, PM2.5, PM10, SO_2_, NO_2_, CO, different observation parameters such as O_3_ change trend in different periods to heat (From: Bai du map).

The data trend analysis changes of the stations in the experimental area include hourly trend analyses, daily average trend analyses, and data trend analyses for each station. The latest collected data are displayed in node charts with high spatial and temporal distributions installed in the area. For the collected data, the air pollution index is calculated corresponding to each collection time based on the calculation method stipulated by its state. The ranking of data collections at multiple points in the region is calculated according to the air pollution index, and the pollution situation of different observation points is analyzed. Based on the installation nodes, the API function of the network map is used to distribute the nodes on the map, and then, the distribution of the collection sites is obtained from the entire area. The following Fig. 3 shows a heat diagram of the change trends of different observation parameters for different dates:

##### Interpolation analysis of air pollutants

The toolbox based on ArcGIS interpolates the observation point data with high spatial and temporal distributions and then obtains the air pollution status of the entire region through limited data collection points. Using the collected data, a different analysis of the regional fine particles, nitrogen and sulfur oxides, the environmental pollution index can be obtained. By using time series data, continuous monitoring of regional environmental pollution can be performed, and by including the monitoring of unexpected data, timely reporting and decision-making for problematic pollution are enabled.

After collecting the different data, we can calculate the data of different places. Via comparison with data from known observation points, we can choose a more suitable interpolation algorithm. By calling the interpolation algorithm interface of the ArcGIS server, we can find an interpolation algorithm with the lowest error for mapping.

#### Data early warning module

**Figure 4:**
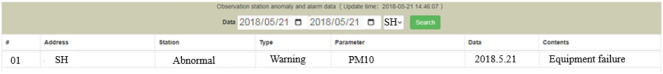
The platform early warning module includes data acquisition anomalies and data threshold anomalies.

The data early warning module is divided into abnormal data collections and abnormal data thresholds. As shown in Fig. 4. The figure shows that the device named SH had a machine failure at 14:46:07 on 2018.05.21.

A data collection anomaly triggers an abnormal alarm if the remote collector receives an abnormal collection (that is, no data are collected); then, the system will push the information to the person in charge, and the appropriate personnel will carry out the specific equipment maintenance.

The principle of the data threshold alarm is to set a specific monitoring threshold for specific pollutants. If the threshold is exceeded, then early warning information is triggered, allowing problematic pollution incidents to be found and treated in time. The data threshold anomaly is divided into two parts. One part makes a judgment according to a set threshold. System administrators can set different early warning thresholds for different pollutants, which will trigger data alarms when the collected data exceed the current threshold (the corresponding situation may be a sudden increase in environmental pollution). The other part is an early warning for different pollutant variation ranges, which are triggered based on early warning information when the collected data increase beyond a certain range. On the whole, the data early warning system mainly relies on the high spatial and temporal distribution data of the wireless sensor networks. This system implements real-time monitoring of pollutant concentration changes for specific observation points in an entire area and processes decisions for sudden high pollution events in a timely fashion.

## Key technologies

### Dual-stack technology

At present, IPv6 technology has not been widely popularized, so this system needs to support IPv4 address access as well as IPv6 address access. Comprehensive comparisons of various IPv6 transition technologies reveal that transitional technologies use dual-stack technology to embed IPv4 protocols and IPv6 protocols into the networking equipment so that routers, servers, and hosts in the network can handle both IPv4 and IPv6 addresses^19,20^.

### Data visualization based on GIS

The visualization of air pollutant data based on GIS technology is based on interpolation analysis of regional data through the spatial analysis functions of GIS and based on the interpolation of the air pollutant concentration distribution map of the entire region using the air pollutant data collected by multiple stations. The spatial analysis technology that GIS mainly uses is the ArcGIS interpolation analysis module, which uses the point-like distributions of atmospheric monitoring station data as a data source to interpolate the concentration of atmospheric pollutants in a target area. The server function of ArcGIS can display online images so that multiple users can access and view them on the Internet^21^. The operation of using ArcGIS to generate a pollution simulation map of the target area is as follows: (a) Use the collected data to calculate the average data of each parameter of each station according to the daily hourly average. (b) Make a shapefile of the target area, and configure the data to fit the base map. (c) Configure observation sites in the target area on the target base map. (d) Bind the observation values of each site in the target area with the corresponding site. (e) Select the data interpolation function of the toolbox, and select the target as the target area of the base map. (f) Click to generate an interpolation fitting graph and select the configuration data to change parameters. (g) Publish the generated fitting image to the target server of ArcGIS Server. (h) Start ArcGIS Server and click to view the published map data. Click the target map to view the air quality fitting map of the target area that has been made.

It is worth noting that when the target site is bound to the corresponding collected data, the data format shown in the following table needs to be used for binding (the data of each site at the same interpolation time). When binding the observation site and the interpolation data, it is necessary to perform unique identification data mapping. In the actual application of the system described in this article, the site name is used for inline mapping. The format of the collected data is shown in Table 4.

**Table 4:** Acquisition data format.

Site	Acquisition time	PM2.5	PM10	SO_2_	NO_2_	CO	O_3_	Temperature	Wind speed
Pioneer park	2018-03-01	24.71	50.84	0	12.3	0.9	57.8	9.64	0.09

**Figure 5:**
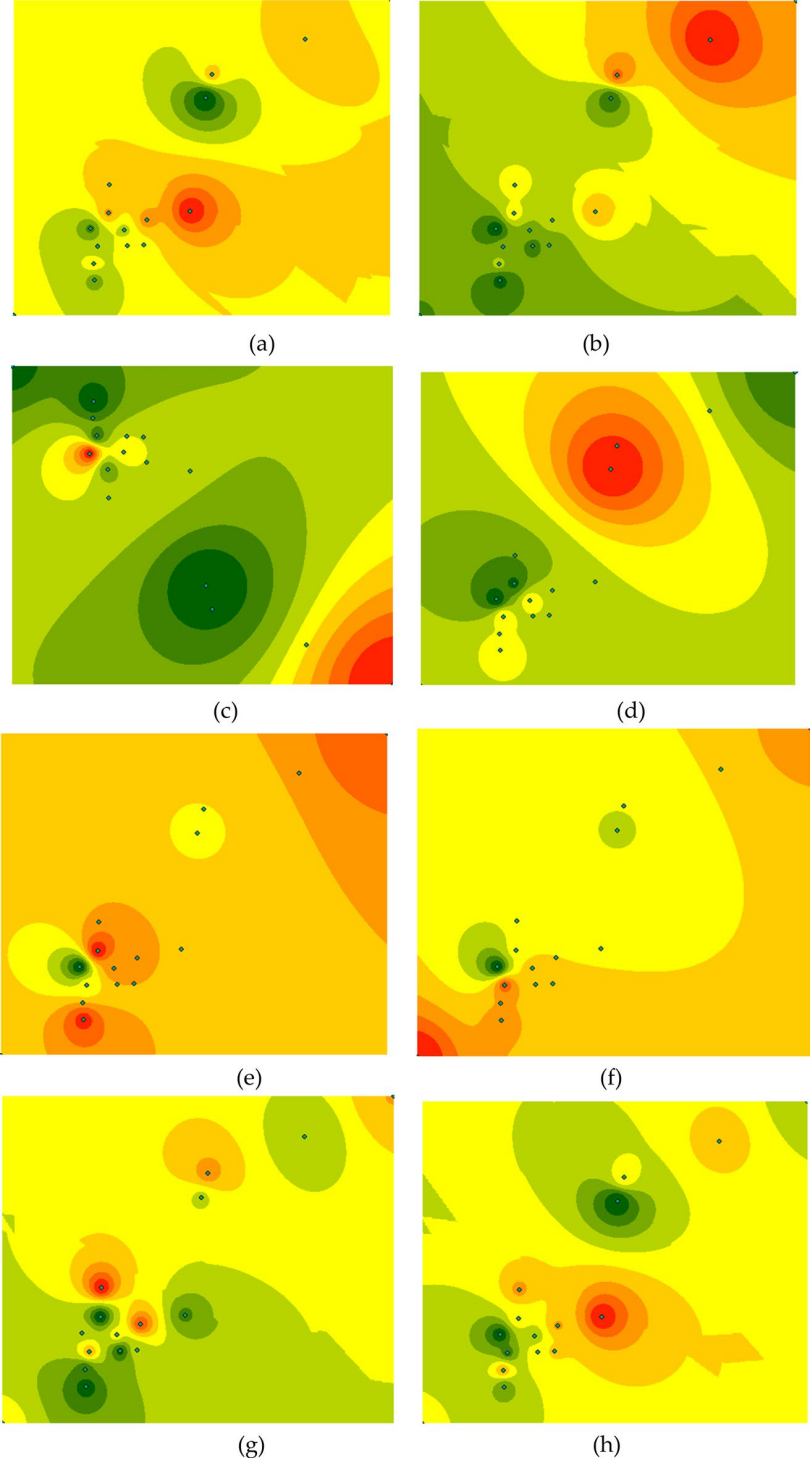
Spatial interpolation of the parameters corresponding to the average value of the data collected on March 3, 2018. (**a**) PM2.5 concentration (**b**) PM10 concentration (**c**) SO2 concentration (**d**) NO2 concentration (**e**) CO concentration (**f**) O3 concentration (**g**) wind speed (**h**) AQI index.

The data collected by the installed data collector are six air quality parameters (PM2.5, PM10, SO2, NO2, CO, O3) and related meteorological factors. When drawing the fitting map of the target area, you can choose to use the air quality index (AQI) obtained by the observation parameters to represent the comprehensive atmospheric environment of the target area, and you can also use different observation parameters to render and calculate the changing trend of different influencing factors in the target area. The data images fitted with different parameters are shown in Fig. 5.

As shown in Fig. 5, different observation sites have different data values, which represent the pollution concentration of the current collection site. If the sites are properly arranged, they can represent the changing trend of the regional atmospheric environment. The use of GIS technology and the high-temporal-accuracy data collected by the wireless sensor network to achieve data fitting obtains the atmospheric environment changes in the target area, and at the same time provides multi-terminal access, which improves the efficiency of data access. Later, it can cooperate with ArcGIS to achieve more data analysis, including the relationship between the terrain analysis of the target area and the atmospheric environment quality, and the relationship between the living area and the non-living area, and the atmospheric environment quality. Provide data interfaces for subsequent research to improve application development efficiency.

The wireless sensor network monitoring station collects daily and hourly data and the background server calculates the daily and hourly average data, which are fitted with the target area as the input shapefile to accompany the corresponding interpolation analysis method in the toolbox, calculate the interpolation fitting diagram, and select the change parameters of the configuration data^22^. Finally, the background publishes the generated fitted image to the ArcGIS server. Users can start the ArcGIS server and click to view the published map data and click the target map to view the air quality fitting map of the target area.

## System testing

### Stability test

**Figure 6:**
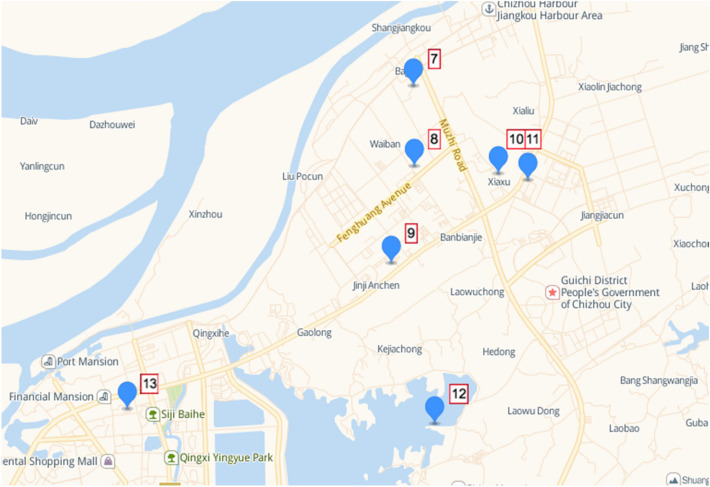
Distribution chart of monitoring points in Chizhou, Anhui Province. (From: Bai du map).

**Figure 7:**
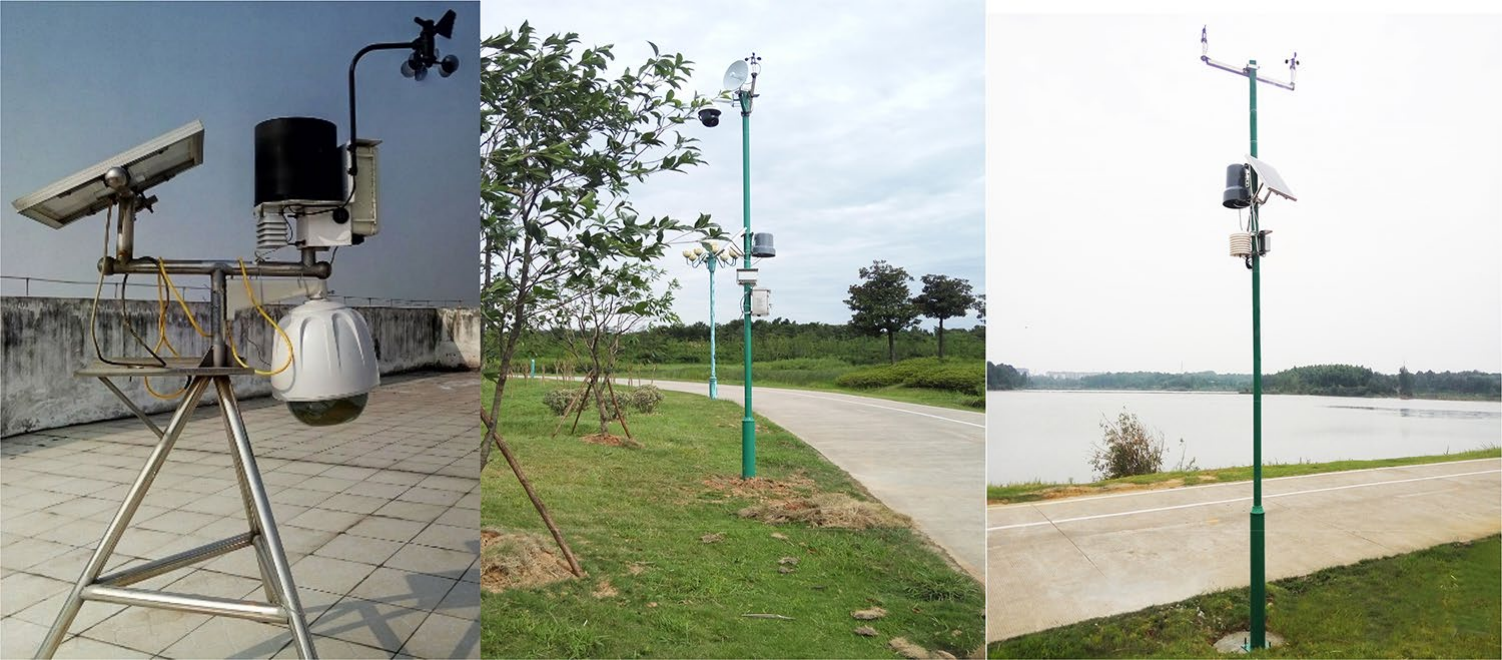
On-site installation equipment pictures.

Taking the experimental station installed in the Chizhou City of Anhui Province as the test area, after the sensors are arranged in the experimental area, the system collects data, draws the current regional pollution status map using historical data, and verifies the performance of the acquisition of real-time high spatial distribution data. The distribution of monitoring points is shown in the following Fig. 6. The picture of the on-site installation equipment is shown in Fig. 7.

Through an analysis of background data, the communication between the wireless sensor terminal node and the central server is determined to work well. The devices are running in the system; the data acquisition is accurate; the data upload is normal; there is no data loss, system downtime, or other negative situations; and the overall network operation is stable.

### Reliability test

**Figure 8:**
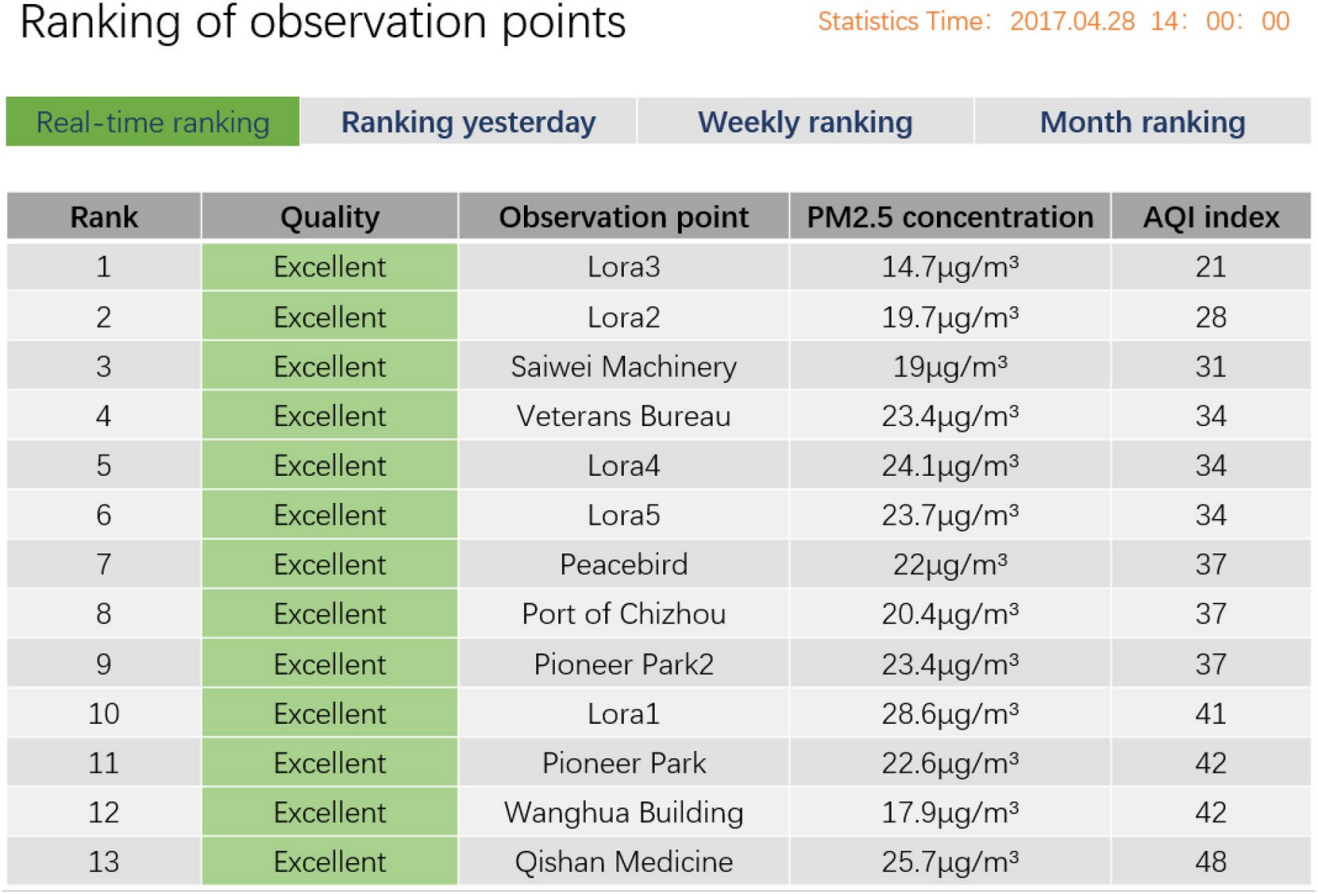
Application module: Ranking of observation points.

**Figure 9:**
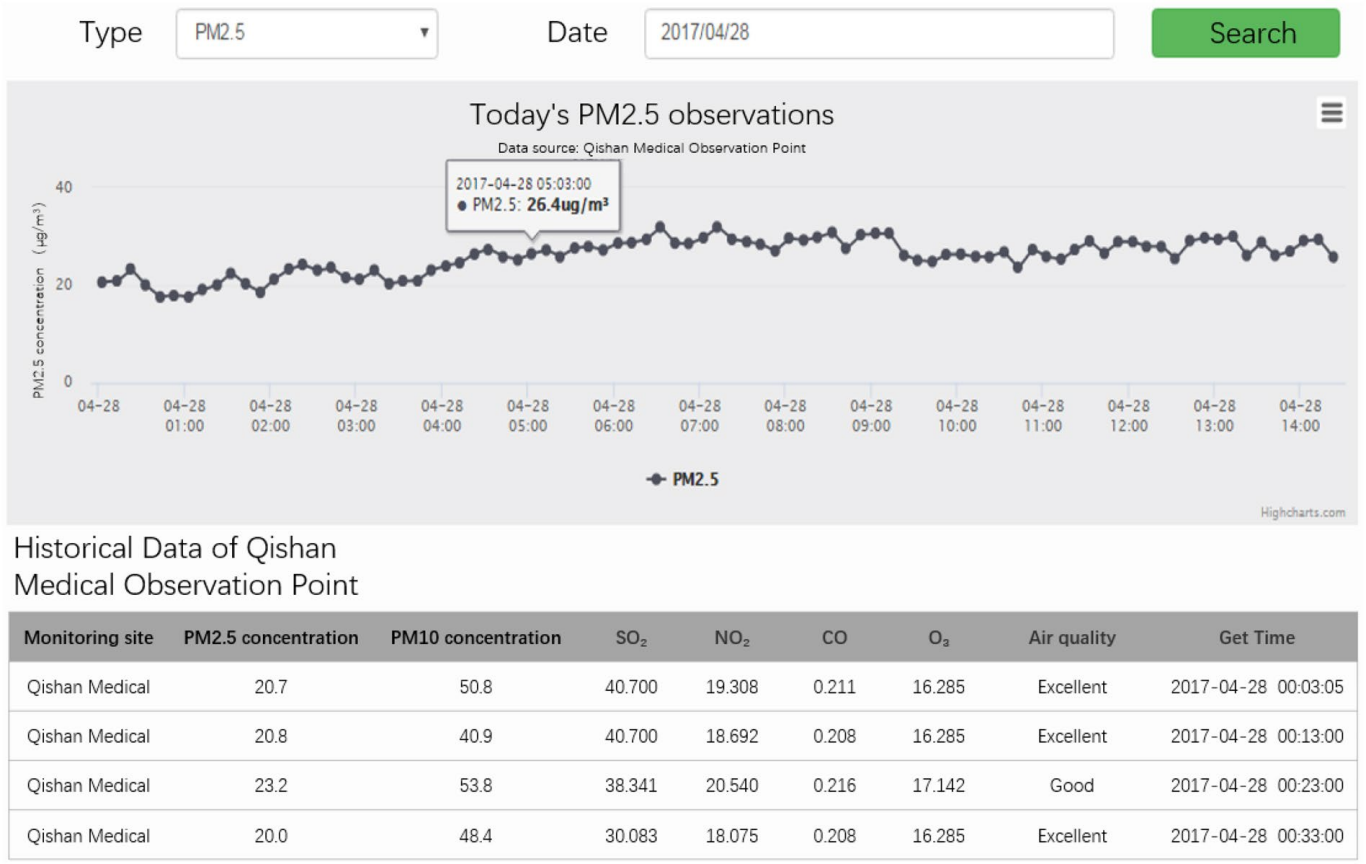
Application module: Historical data.

The operational reliability of the system is evaluated from five aspects: real-time data acquisition verification, the feasibility of multipoint and multi-time series regional environmental pollution analysis and mapping, pollutant threshold alarm verification, sudden pollutant growth verification, and availability of historical data analysis of the observation points. The application module is shown in Figs. 8 and 9.

The system runs for each acquisition device, enabling real-time visualization of the data collected by the device. Users can view the latest 24-h data and 30-day average data of the current device, see the changing trends and evolution of the data by means of a map interface, display the pollution degrees of the data by means of a heat map, and view the data evolution of different scales for different parameters in different time periods. In addition, during the operation of the system, when collecting data, we can view the current early-warning events in the function module that are based on the warning strategy set by the system.

After verification, the air pollution monitoring system can update in minutes, basically matching the monitoring frequency of real-time monitoring systems. The fitting analysis of the overall environment of the region conforms to the overall actual situation of the region, and the current overall environmental quality data of the region can be obtained through multipoint monitoring points. An early warning analysis of abnormal data collections and pollution statistics can successfully deliver the expected analysis. An analysis of the historical environmental data of the observation points can also provide results in a limited time, and the main innovation of our system is the processing of background data. On the whole, the system executes the expected functions and provides high-precision monitoring of air pollutants.

### Performance test results

After performance testing, the system can accurately locate the location of the collection device through geographic location information. It also provides an overview of the current equipment data. You can view the latest data of all installed equipment. Through tabular data and single-site data, we can calculate and analyze the real-time air quality, AQI, primary pollutants, and air level of each collection site. According to the early warning strategy set by the system, when the collected data trigger the early warning rules, you can view the current early warning events. The system also provides downloading of full data and statistical calculation of real-time data.

### Users feedback

During the software trial operation phase, internal user feedback was good. The first-level function data visualization module, data early warning module, background data management function module, and other functions operate normally. The secondary functions of the collection equipment location management module, equipment real-time data acquisition module, equipment latest data display module, atmospheric air quality analysis module, air warning list module, data download, and analysis module, document management module, and other functions are operating normally. According to user feedback, the air pollutant monitoring and early warning platform have achieved the expected functions, but the fluency of human–computer interaction still needs to be improved. The function and function descriptions of the system are shown in Table 5.

**Table 5:** The function and function description of the system.

Level 1 function: data visualization module	
Secondary function	Function description
Collection equipment location management	The location information of the data collection device is displayed through the geographic location information, which can accurately locate and provide an overview of the current device data
Real-time data acquisition of equipment	For each collection device, real-time visualization of the data collected by the device is realized. The user can view the current device's data for the last 24 h and the 30-day daily average data, and provide two ways of data curve and data table
Latest data of all devices	You can view the latest data of all installed equipment, display the data in the form of a list, and enter the data of a single site through the site name
Atmospheric air quality analysis	Calculate and analyze the latest air quality of the latest collected data of each collection site, calculate AQI, primary pollutants, air grade, and quality
Air warning list	According to the early warning strategy set by the system, when collecting data and starting the early warning rules, you can view the current early warning events in this function module
Air warning list	
Equipment management	Provide the administrator to view the information of the installed equipment to facilitate even the equipment maintenance
Warning list and rule management	The provided early warning list is consistent with the data provided to the public, and statistics are made on the triggered early warning events. Early warning rule management, providing rule configuration function, can configure the early warning detection rules for drinking according to different needs
Data download and analysis	Provide full data analysis and download, provide a download for historical data, and perform statistical calculations on real-time data
Document management	For the management of information documents released by the front desk, including operations such as creating and deleting documents

## Conclusion and future works

The highly accurate spatiotemporal monitoring and early warning platform for air pollutants based on IPv6, as implemented in this paper, solves the problem of IP address shortages for wireless sensors by using IPv6 technology, and the data transmissions are safer and more efficient. The system adopts a Spring MVC framework and implements many functions, such as air pollutant data collection, data visualization, and data early warning. Combined with the ArcGIS interpolation algorithm, the air pollutant concentration status of the entire region is calculated, and the interface is established at an early stage of development. In a later stage, the data prediction function is implemented by combining machine learning and other related algorithms, which provides more powerful decision support tools for air pollution monitoring. The system was deployed in Chizhou City, Anhui Province, to perform a field evaluation. After testing, the efficiency and accuracy of the system met the requirements for air pollutant monitoring, and the system ran stably and had definite reliability. The specific work is as follows:

1. Applied research on regional environmental monitoring systems based on wireless sensor network technology.
2. The communication mechanism between the terminal collector and the central server is established, and the two-way communication of data is realized through the communication method based on the TCP protocol, which ensures that the central server data instructions are issued. Take advantage of the real-time communication of the computer to realize the real-time control of the collection terminal;
3. Established a basic functional platform for monitoring data visualization based on B/S architecture and GIS technology. The B/S architecture ensures that the data terminal can be viewed by multiple users. Using the characteristics of the browser, it can realize the collection and processing of single data terminal and multi-terminal data. View the effect. At the same time, the MVC method is used to realize the separation of data and operation, and to ensure the stability of data. At the same time, GIS technology is used to achieve high spatial precision data interpolation and then analyze the environmental changes of the entire region, and compare the environmental quality changes of the multi-objective observation area. Realize regional grid-based environmental monitoring, and achieve near-real-time physical examination of the regional environment;
4. Using the collected data with high temporal and spatial precision distribution, the relationship between the target observation factor and environmental quality is established by analysis. Using the artificial neural network model, analyze and establish the distribution of the influence weight of each input parameter and target parameter, obtain the core pollution parameters, and provide a data basis for specific environmental management and control. After analysis, it is found that the concentration of fine particles has a significant impact on air quality and is directly proportional, while other inhibitory factors, such as wind speed and rainfall, will improve air quality. The data model of integrated learning can accurately simulate the relationship of various parameters, which is of reference significance for the prediction of air quality.
5. Utilizing the high spatiotemporal accuracy of the wireless sensor network, it can realize near real-time data collection of the regional environment, and real-time data statistics can be realized for the sudden changes of environmental factors in a specific observation area. When abnormal data changes occur in a specific observation area, you can pass Configure early warning rules on the central server to realize sudden alarms of data. The alarm rules designed by the system include data threshold alarm, data mutation alarm, and terminal collector data collection failure alarm. Through the preset alarm rules, when the collected data triggers the corresponding rules, the relevant responsible persons can be notified through real-time communication means (email, SMS, etc.) to complete the timely handling of sudden environmental changes in the target observation area.

The data used by the system mainly point data collected by wireless sensors, and then use the interpolation analysis technology of ArcGIS to perform interpolation analysis on point data, extend the points to area data, and provide environmental management departments with regional environmental governance and management Data support.

In the future, the system will also incorporate multiple data such as remote sensing data inversion products, vehicle-mounted, and hand-held monitoring data. Among them, remote sensing inversion products can only provide data during the day and lack more complex night data. The night is a key requirement for secret release. The time of interest. It is difficult to guarantee the timeliness of remote sensing inversion products. Vehicles are generally used for emergency monitoring, and the accuracy of the handheld cannot be guaranteed. Therefore, the fusion and application of multiple data will be a difficult point to be solved. In addition, the system will also introduce the results of prediction and early warning research based on machine learning and use multi-source data fusion to provide future hourly and day-based forecast and early warning functions. The related work of the above platform expansion can be used for atmospheric environment Precise governance, management, and data support for public travel arrangements are all meaningful tasks.
